# Hantavirus Pulmonary Syndrome , Southern Chile, 1995–2012

**DOI:** 10.3201/eid2104.141437

**Published:** 2015-04

**Authors:** Raúl Riquelme, María Luisa Rioseco, Lorena Bastidas, Daniela Trincado, Mauricio Riquelme, Hugo Loyola, Francisca Valdivieso

**Affiliations:** Universidad San Sebastian, Hospital de Puerto Montt, Puerto Montt,Chile (R. Riquelme, M.L. Rioseco, M Riquelme);; Hospital de Puerto Montt, Puerto Montt (L. Bastidas, D. Trincado);; Clínica Alemana Universidad del Desarrollo, Santiago, Chile (H. Loyola, F. Valdivieso)

**Keywords:** hantavirus, Andes virus, Chile, hantavirus pulmonary syndrome, vector-borne infections, viruses

## Abstract

Hantavirus is endemic to the Region de Los Lagos in southern Chile; its incidence is 8.5 times higher in the communes of the Andean area than in the rest of the region. We analyzed the epidemiologic aspects of the 103 cases diagnosed by serology and the clinical aspects of 80 hospitalized patients during 1995–2012. Cases in this region clearly predominated during winter, whereas in the rest of the country, they occur mostly during summer. Mild, moderate, and severe disease was observed, and the case-fatality rate was 32%. Shock caused death in 75% of those cases; high respiratory frequency and elevated creatinine plasma level were independent factors associated with death. Early clinical suspicion, especially in rural areas, should prompt urgent transfer to a hospital with an intensive care unit and might help decrease the high case-fatality rate.

## Abstract 2

Early clinical suspicion should prompt urgent transfer of patients to a hospital with intensive care facilities.

Since the first cases described in United States in 1993, hantavirus pulmonary syndrome (HPS) has been reported in the United States, Argentina, Bolivia, Brazil, Chile, Ecuador, Paraguay, Panama, Uruguay, and Venezuela (*1*). Several types of New World hantaviruses (family *Bunyaviridae*) have been recognized. Their distribution is determined by the density of rodent populations serving as specific reservoirs of each virus type.

In Chile, Andes virus is the only identified hantavirus (*2*). It was first reported in 1995 during an outbreak in Argentina and is carried by the murid rodent *Oligoryzomyslongicaudatus* (i.e., long-tailed mouse or “colilargo”) in southern Argentina and central and southern Chile (*3*).

In Chile, where HPS is subject to immediate mandatory reporting to health authorities, a total of 786 cases occurred during 1995–2012. Regional and seasonal incidences varied from 0.17 to 0.53 cases per 100,000 inhabitants (*4*). Despite such low incidence, HPS is of public health concern because of its severity and its high case-fatality rate (CFR) (20%–60%).

We examined the clinical and epidemiologic features of HPS during 17 years in the provinces of Llanquihue and Palena, which had the highest incidences of this disease in Chile. This geographic area is served by the Health Service of Reloncaví (HSR) in Puerto Montt city, which has its 420-bed reference center at the Hospital of Puerto Montt in Puerto Montt.

## Material and Methods

### Study Site and Population

**Figure 1:**
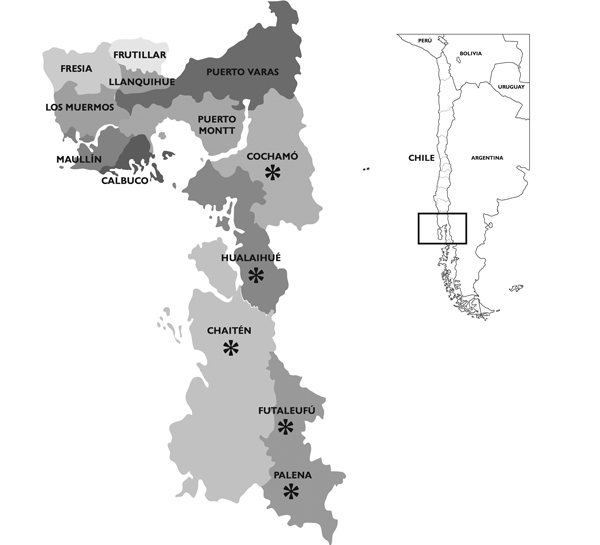
The 13 communes in the provinces of Llanquihue and Palena, southern Chile. (Two communes share the name of the province to which they belong.) Asterisk indicates Andean communes. Inset: South America, with study area in box.

The provinces of Llanquihue and Palena are located in southern Chile, on the western edge of South America. Together they comprise 30,178 km^2^ and 340,464 inhabitants. These 2 provinces are subdivided into 13 communes (Figure 1).

Our study comprised all HPS cases reported to HSR during 1995–2012. All were confirmed by serologic tests performed at the National Reference Centers at the Public Health Institute (Santiago) or Universidad Austral (Valdivia). These tests are ELISAs for IgM and IgG that use hantavirus Sin Nombre antigen provided by the US Centers for Disease Control and Prevention (Atlanta, GA, USA).

### Data Collection

We obtained data from 3 sources. First, we used epidemiologic records from all cases reported during 1995–2012. Data included patient age, sex, occupation, residence, site of probable infection, contact with other HPS patients, dates of hospitalization, and outcome.

Second, we reviewed clinical records of all patients admitted to Hospital of Puerto Monttwith confirmed HPS during the same period. Data recorded were age, sex, probable mechanism of infection, incubation period (only for patients for whom precise information about the time of rodent exposure and onset of symptoms was available), and medical history. On admission, presence of dyspnea, fever, asthenia, headache, myalgias, chills, cough, abdominal pain, and cyanosis and blood pressure, pulse, temperature, and respiratory frequency were recorded. During hospital stay, the following data were collected: presence of bleeding, alterations in renal and hepatic functions, admissions to intensive care unit (ICU), oxygen support, arterial oxygen tension/inspiratory oxygen fraction (PAFI) index, steroid administration, mechanical ventilation (MV) (specifying timing of connection), and circulatory shock. Shock was defined as systolic blood pressure <90 mm Hg that did not improve with fluid administration or that required the use of vasoactive drugs and abnormalities in tissue perfusion manifested by alteration of consciousness, oliguria, and lactate acidosis (*5*). One of the authors (R.R.) analyzed chest radiographs and classified the infiltrates as alveolar, interstitial, or mixed pattern and unilateral or bilateral; number of compromised quadrants and presence of pleural effusion were recorded. Results of laboratory tests performed on admission and during illness were also recorded. Each case was classified into 1 of 3 groups: grade I (mild disease) when patients had only prodromal symptoms without pulmonary involvement; grade II (moderate disease) when patients had interstitial pulmonary infiltrates or required supplemental oxygen but were hemodynamically stable; and grade III (severe disease) when patients required MV or had hemodynamic instability (*6*). Final outcome (death or survival) was also recorded.

Finally, we reviewed reports of epidemiologic inspections to homes, workplaces, and probable sites of infection (dwellings and their surroundings) at the time of case report to HSR. A survey administered to each patient or to close relatives asked about HPS risk during the 6 weeks before symptom onset. Visited places were classified as urban, rural, or semirural. We stratified the infection risk in visited dwellings according to a 5-parameter scale, each with 1 point assigned to absence of foundations, presence of holes, poor ventilation and lighting, presence of trash without adequate container inside the dwelling, and grainstorage, flour, and other food packaging Risk was considered high for scores 4–5, moderate for 2–3, low for 1, and absent for 0. We similarly classified dwelling surroundings according to presence of droppings, rodent pathways, rubbing stains, gnawing signs, rodent nests or holes, or observation of rodents themselves.

### Statistical Analyses

We used the Student *t* test to compare parametric variables and χ^2^ and Fisher exact tests to compare discrete variables when necessary. A p value <0.05 was considered statistically significant. Incidence rate of HPS per commune was calculated from information provided by Chilean Census 2002 (*7*).

## Results

During 1995–2012, a total of 103 confirmed HPS cases were reported to HSR. Mean age of patients was 35 ± 17 years (range 3–80 years); 71 (69%) were men. Overall CFR was 32% (33/103); CFR for the 80 HPS patients admitted toHospital of Puerto Montt was 30% (24/80).

### Epidemiologic Characterization

We identified 52 rural locations as probable infection sites for 100 patients. For the remaining 3 patients, infection site could not be determined because of exposure to several risky sites.

Infection most likely was acquired through farming and forestry work for 44% of patients and was associated with recreational activities for 13%. For the remaining patients, infection-associated activity was not determined because of similar risk at home and at work.

HPS incidence per 100,000 inhabitants varied widely among communes. The highest rates occurred within Andean mountainous areas, mainly Palena and Cochamó communes (350 and 364 cases per 100,000 inhabitants, respectively). Incidence for the aforementioned communes was 8.5 times higher than that for the rest of the region (Table 1; Figure 1).

**Table 1:** Incidence of hantavirus pulmonary syndrome, Health Service of Reloncaví, Chile, 1995–2012

Commune	No. patients, n = 103	Population*	Incidence rate†
Cochamo‡	16	4,399	363
Palena‡§	6	1,715	350
Chaiten‡	12	7,290	164
Fresia	10	12,861	77
Los Muermos	13	17,004	76
Futaleufu‡	1	1,849	54
Maullin	4	15,205	26
Calbuco	8	32,792	24
Hualaihue‡	2	8,464	24
Frutillar	3	16,504	18
Puerto Varas	6	35,590	16
Puerto Montt	19	196,561	10
Llanquihue§	0	17,228	0
Unknown	3	Not applicable	Not applicable
Andean area	37	23,717	156
Not Andean area	63	343,165	18

*Census 2002 (http://www.ine.cl/cd2002/). †Per 100,000 inhabitants. ‡Communes of Andean area. §Palena and Llanquihue communes have the same name as the province to which they belong.

**Figure 2:**
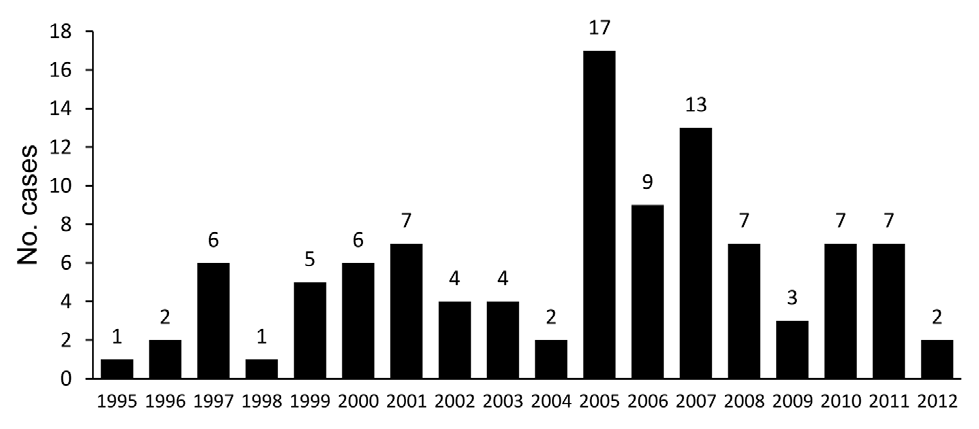
Number of hantavirus pulmonary syndrome cases in provinces of Llanquihue and Palena, southern Chile, 1995–2012.

**Figure 3:**
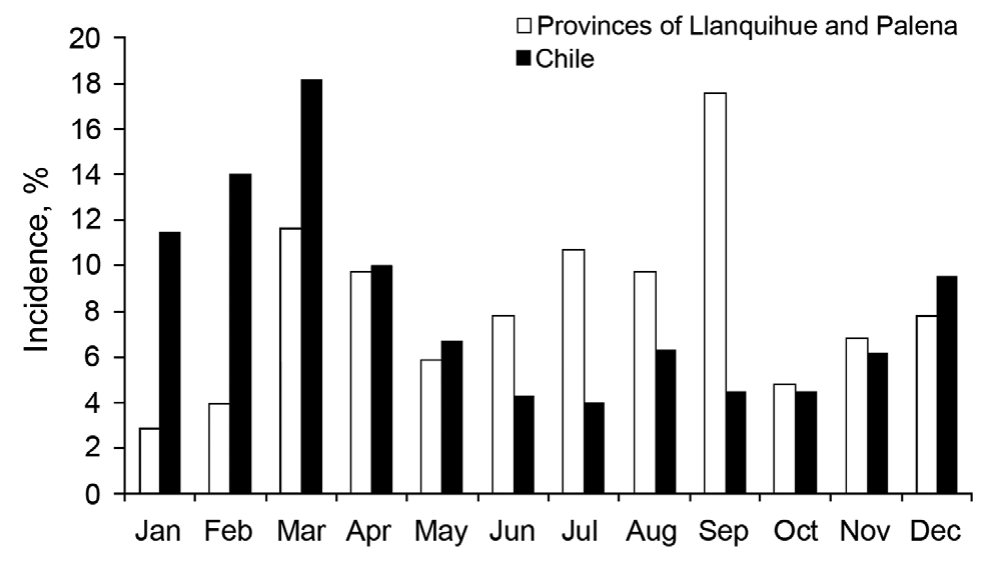
Seasonal incidence of hantavirus pulmonary syndrome, provinces of Llanquihue and Palena, Chile (n = 103), and entire country (n = 785), 1995–2012.

Yearly incidence varied widely during 1995–2012. Most cases occurred during 2005–2007 (Figure 2). In the region studied, incidence was highest during winter. By contrast, in the rest of the country, incidence was highest during summer and autumn (Figure 3).

For 23 patients, HPS occurred in related persons and made up a total of 8 clusters, 5 with 3 cases each and 4 with 2 cases each. All patients in each cluster shared both environmental risk factors and family relationship; included in the clusters were 6 cohabitating couples.

Epidemiologic reports on home or workplaces were available for 42 patients. Thirty-one percent of houses, 43% of housing environments, and 39% of working environments had high or moderate risk for rodent infestation.

### Clinical Characteristics

The 80 HPS patients admitted to Hospital of Puerto Montt during 1995–2012 represented 78% of cases reported to HRS during that period. Seventy percent were men. Patients were 35.7 ± 16 years of age (range 4–73 years), and 90% were rural inhabitants. Their main occupational activities were farming or forestry (35%), housework (25%), student (15%), and fishery or marine harvesting (11%). Of these patients, 25 (31%) had a history of contact with rodents or rodent droppings. Infection was attributed to occupational exposure for 35 (44%) patients and traveling to a high incidence zone for 10 (12%).

Mean interval from appearance of symptoms to hospitalization was 5.7 ± 3 days (range 2–17 days). Incubation period, estimated from the analysis of 20 patients, was 10 ± 7 days (range 2–28 days).

For patients admitted during 1995–2004, HPS was considered 1of the admission diagnoses for only 7 (27%) of 26 case-patients. This presumptive diagnosis increased to 76% (37/49) during 2005–2012 (Tables 2, 3).

**Table 2:** Clinical characteristics on admission to Hospital Puerto Montt for 80 patients with confirmed hantavirus disease, Puerto Montt, Chile, 1995–2012

Symptoms/signs	No. (%) patients
Main symptoms	
Fever	73 (91)
Myalgia	57 (71)
Headache	39 (49)
Respiratory distress	36 (45)
Abdominal pain	35 (44)
Cough	32 (40)
Malaise	31 (39)
Vomiting	19 (24)
Diarrhea	17 (21)
Anorexia	17 (21)
Chills	8 (10)
Rare symptoms	
Generalized rash	1 (0.1)
Low back pain	4 (0.5)
Bloody sputum	2 (0.3)
Confusion	2 (0.3)
Sore throat	1 (0.1)
Urinary	1 (0.1)
Signs on chest radiograph	
Infiltrates	75 (94)
Interstitial pattern	53 (66)
Alveolar pattern	12 (15)
Mixed pattern	10 (13)
Bilateral	71 (95)
Infiltrates in 4 quadrants	39 (49)
Opacity progress by ≥50% within 48 h	24 (30)

**Table 3:** Vital signs and laboratory results for 80 hantavirus pulmonary syndrome patients admitted to Hospital Puerto Montt, Puerto Montt, Chile, 1995–2012

Parameter	Median ± SD (range)
Laboratory value*	
Hematocrit, %, n = 74	47 ± 7 (32–72)
Platelets × 10^3^/μL, n = 75	67,505 ± 42,717 (14,000–238,000)
Leukocytes × 10^3^ cells/μL, n = 74	13,520 ± 9,971 (2,800–59,400)
Creatinine, mg/dL, n = 70	1.41 ± 0.97 (0.6–6.6)
Bilirubin, mg/dL, n = 64	0.62 ± 0.70 (0.03–5.9)
Aspartate aminotransferase, U/L, n = 66	245.2 ± 178.2 (26–705)
Alanine aminotransferase, U/L, n = 44	172.2 ± 134.7 (11–536)
Prothrombin, %, n = 56	81.1 ± 22.4 (13–100)
Vital signs
Temperature, °C, n = 75	37.7 ± 1.1 (35–40.5)
Pulse, beats/min, n = 76	109,13 ± 22(61–158)
Respirations, breaths/min, n = 63	30.6 ± 9.5 (16–60)
Systolic blood pressure, <90 mm Hg, n = 79	22.5 %

*Reference values are as follows: hematocrit 37%–47%, platelets 140–440× 10^3^/μL, leucocytes 4,1–10.9× 10^3^ cells/μL, creatinine 0.5–0.9 mg/dL, bilirubin 0.05–1.0 mg/dL, glutamic-oxalacetic transaminase 10–50 U/L, glutamic-pyruvic transaminase <35 U/L, prothombin 70%–120%.

Duration of hospitalization for HPS patients was 5.2 ± 4.6 days (range 1–25 days). Sixty-three (79%) case-patients were admitted to the ICU; 72 (90%) required oxygen administration; and 40 (50%) were connected to MV for 4.2 ± 4.7 days (range 1–17 days).

Mean PAFI at admission was 216 ± 107 (range 40–508). Five (6%) patients had no pulmonary involvement. Shock occurred in 37 (46%) patients, all of whom received vasoactive drugs as prescribed. Hemorrhagic manifestations occurred in 31 (39%) patients: hematuria in 15 patients; cutaneous or puncture sites bleeding in 12 patients, hemoptysis in 5 patients, metrorrhagia in 4 patients; and epistaxis gingivorrhagia, rectorrhagia, and epidural hematoma after lumbar puncture in 1 patient each.

For 95% of patients, platelet counts were <100 × 10^3^/μL(reference range 140–44010^3^/μL) at a given time; for 34%, platelet counts were <35 × 10^3^/μL. The mean platelet count was 50 ± 39 (range 8–238) × 10^3^/μL. In 48% of hospitalized patients, creatinine increased >1.2 mg/dL (reference range 0.5–0.9 mg/dL); 4 (5%) of these patients required hemodialysis. Hepatic enzymes were elevated in 57 (71%) patients.

Thirty-one patients received steroids. Methylprednisolone was administered to 20 patients in accordance with a published protocol (*8*): 1 g intravenously per day for 3 days, followed by 16 mg orally per day for 3 days, 8 mg per day for 3 days, and 4 mg per day for 3 days. Eleven other patients were enrolled in a clinical trial and received methylprednisolone 1 g intravenously per day for 3 days (*9*). Nevertheless, we observed no difference in CFR between patients who did and did not receive methylprednisolone.

According to their clinical course, 5 (6%) patients were classified as having grade I HPS; 34 (42%) as having grade II HPS, and 41 (51%) as having grade III HPS. Twenty-four (30%) hospitalized patients died; for 21 (88%) of these, death was attributed directly to HPS. Shock was considered the cause of death for 18 (75%) patients, respiratory failure for 2 (8%), multiorgan failure for 2 (8%), and secondary sepsis for 2 (8%) (1 gram-negative sepsis and 1 *Staphylococcus aureus* sepsis).

Fifteen (63%) of 24 patients died during the first 24 hours after admission, and 22 (92%) died during the first 72 hours after admission. All deaths occurred among patients with grade III disease. Independent factors associated with death were respiratory frequency >30 breaths/minute and creatinine >1.3 mg/dL on admission (Table 4). CFR did not differ by sex, abnormal hepatic test results, or chest radiographic images progression >50% in 48 hours. Furthermore, we found no relation to CFR for patients with hematocrits >45% or >50%; platelet counts <100, <50 or <35 × 10^3^/μL at admission; or PAFI <250, <200, <150, or <120 at admission.

**Table 4:** Study variables with significant differences between hantavirus pulmonary syndrome patients who did and did not survive, Hospital Puerto Montt, Puerto Montt, Chile, 1995–2012

Variable	Survivors, no. (%), n = 56	Nonsurvivors, no. (%), n = 24	Odds ratio (95% CI)	p value
Systolic blood pressure <90 mm Hg*	8 (14)	10 (41.7)	4.0 (1.3–12.1)	0.014
Respirations >30 breaths/min*	13 (23)	19 (79.2)	15.3 (3.8–61.5)	0.000
Pulse >120 beats/min*	14 (25)	14 (58.3)	4.0 (1.4–11.1)	0.008
Bleeding manifestations	15 (27)	17 (70.8)	6.8 (2.3–19.7)	0.000
Creatinine >1.3 mg/dL*	13 (23)	13 (54.2)	3.7 (1.2–10.6)	0.017
Admitted to intensive care unit	39 (70)	24 (100)		0.001
Mechanical ventilation	16 (29)	24 (100)		0.000
Shock	13 (23)	24 (100)		0.000
Infiltrates in 4 quadrants in chest radiograph*	21 (38)	18 (75)	5.0 (1.6–15.5)	0.006
PAFI <100*	2 (4)	5 (20.8)	7.1 (1.2–41.2)	0.008
Classified as grade III (severe)	17 (30)	24 (100)		0.000

*On admission. PAFI, arterial oxygen tension/inspiratory oxygen fraction.

## Discussion

HPS is endemic in southern Chile, and human–rodent contact is considered the main mechanism for transmission. Several factors can explain the occurrence of human HPS in each of the 17 years of this study; these include a favorable habitat for rodent populations, which enables circulation of the virus between them (*10*), and high percentages of rural population (Llanquihue 27.5% and Palena 60%) for whom agriculture and forestry as the main occupational activities. Consequently, humans invade the rodents’ natural habitat, the temperate rain forest and its residues. Epidemiologic visits also found evidence of inadequate rural housing and peridomestic and workplace conditions that permitted rodent invasion; for 87% of patients, those places were considered the probable infection site. These findings reflect the usual conditions of life of the rural population and provide evidence that humans are exposed to hantavirus at home and in their workplaces. This contact is expected to increase if rodent population augments.

The incidence of cases varied in time. In Chile and southern Argentina, disease incidence has increased coincidence with the synchronic flowering and seeding of the shrub *Chusqueaquila*, a perennial bamboo that occurs in long inter annual cycles and provides abundant food for the granivorus rodent *O. longicaudatus*. Large outbreaks of rodents (known as “ratadas”) are associated with this cyclic phenomenon (*10*, *11*), with existing chronicles as old as the conquest and Spanish colonization of the country (*12*).

In Chile, reported cases peaked in 2001, but in our study, cases peaked in 2005–2007. A possible explanation for the increase in HPS cases during 2005–2007 (Figure 3), without evidence of “ratada,” is that other native trees (olivillo, *Aextoxiconpunctatum*; avellano, *Gevuinaavellana*; and others), shrubs (“murtilla”), and herbs also have cycles of seed production not clearly known because they are not under surveillance and therefore not reported. These seasonal cycles could be associated with localized and minor differences in rodent population density (*13*). More research on the characteristics of the reservoir species and its habitat is needed to better understand the epidemiology of human disease

In the United States, HPS displays a strong seasonal distribution. Most cases occur in May, June, and July; the fewest occur in December, January, and February (*14*). In Chile, HPS incidence is also highest during the Southern Hemisphere summer (*4*) (Figure 3); the same has been described in Argentina (*15*). During previous years, brief periods of observation found this seasonal distribution in the study region (*10*). Seasonal distribution has been suggested to be a consequence of increasing human recreational and occupational activities in rural areas (*10*,*16*). However, we found that most cases occurred during autumn and winter, in coincidence with the increase in *O. longicaudatus* mice, resulting from increased seed availability. Otherwise, this rodent goes out into open spaces in summer to reproduce, favoring human contact on vacations and outdoor activities (*17*).

In our study, the increase in HPS cases during autumn and winter suggests a particular form of contagion. In the provinces studied here, humans live and work in the invading rodents’ habitat during times when rodents are more abundant.

Hantavirus seroprevalence in rodents varies by season and geography. Captures in our region during 1998–2001 showed seroprevalence rates of 7.2%–13.5%, which is higher than in the rest of the country (1.5%–3.2%). This prevalence could be even larger in *O. longicaudatus* mice captured in a patient’s home and peridomestic storage buildings (*10*).

The native landscape fragmentation caused by forestry and agriculture has favored the overgrowth and wider distribution of *C. quila* bamboo and the movement of *O. longicaudatus* mice between patches of vegetation, which increases the chance of human–rodent contact (*18*,*19*). This observation could explain the higher incidence of HPS in communes of our region exhibiting higher densities of *C. quila* bamboo and in the Andean region next to El Bolson, Argentina, where the first HPS cases in South America were reported, followed by the communes of Los Muermos and Fresia in the Cordillera de la Costa (Table 1; Figure 1).

Since identification of the first cases of HPS in HSR, clinicians have improved their initial diagnostic accuracy from 27% during 1995–2004 to 76% during 2005–2012. Accurate diagnosis is important because HPS is an unusual disease, even in a zone to which it is endemic, and early suspicion enables timely and effective management.

The disease characteristics we observed—fever, myalgias, thrombocytopenia, increased hematocrit, leukocytosis, and elevated creatinine, followed by different degrees of pulmonary involvement, usually with rapidly evolving acute respiratory distress—confirm what we described previously among 25 cases (*6*) and matches HPS descriptions from the United States (*14*,*20*). However, in the US cases, hemorrhagic manifestations were not described; in Chile, hemorrhagic manifestations have been repeatedly reported (*6*,*8*,*21*,*22*), even though in this study they were less frequent than in 2003 (39% vs. 64%, respectively). The 32% CFR is similar to that reported in the United States (35%) (*14*).

On the basis of an open study suggesting the benefit of high-dose steroids for HPS (*8*), we administered that dose to 31 of the patients reported here. We discontinued high-dose steroids after a controlled trial showed its failure to decrease the severity of HPS (*9*).

In this study of a large number of cases, we confirmed the variable characteristics of hantavirus disease, from only mild prodromal symptoms without cardiopulmonary involvement to the severe cardiopulmonary syndrome, as observed in a small number of cases studied previously in our region (*6*). Five patients in this study showed no evidence of pulmonary involvement, which is consistent with seroprevalence studies identifying hantavirus-seropositive persons without history of severe disease in up to 10% of some groups of Andean inhabitants in Chile (*23*). Reports on hantavirus seropositivity in persons without pulmonary involvement also have been presented in Argentina and the United States, although at lower frequencies (*14*,*15*). As long as pulmonary symptoms are required for reporting of hantavirus infection to the US Centers for Disease Control and Prevention, these milder hantavirus infections will continue to go uncounted (*14*). Causes for different clinical characteristics of hantavirus infection are under investigation and can be related to the virus, the human host, or both (*24*).

Severe hantavirus disease is characterized by a rapid installation and progression of severe respiratory failure and shock, which requires urgent ICU management, which is not always available in a timely manner. Death occurs in most cases within 1–2 days after hospital admission. Respiratory frequency >30 breaths/minute and creatinine levels >1.3 mg/dLon hospital admission were independent factors associated with death (Table 3).

In US studies, platelet count was significantly lower in patients who died than in those who survived, but we did not confirm this finding. Low platelet count and high hematocrit are good indicators for suspecting the diagnosis, but we did not correlate them with death. The presence of these 2 elements with compatible clinical and epidemiologic background should prompt rapid transfer of the patient to a hospital with ICU facilities. A rapid test to detect hantavirus IgM based on recombinant N-protein of Puumala virus (IgM POC PUUMALA, Reagena Ltd, Toivala, Finland) was evaluated for Andes virus diagnosis and showed >90% sensitivity and specificity It is available in Chile and is of help in some cases for decision making (*25*). Early clinical suspicion of hantavirus disease, especially in small rural areas, must indicate urgent transfer to a hospital with ICU and may help decrease the high CFR observed in patients with HPS.
